# Advances in understanding the role of adipose tissue and mitochondrial oxidative stress in *Trypanosoma cruzi* infection

**DOI:** 10.12688/f1000research.19190.1

**Published:** 2019-07-22

**Authors:** Jyothi F. Nagajyothi, Louis M. Weiss

**Affiliations:** 1 Department of Microbiology, Biochemistry and Molecular Genetics, Public Health Research Institute, New Jersey Medical School, 225 Warren Street, Newark, NJ, 07103, USA; 2 Departments of Pathology and Medicine, Albert Einstein College of Medicine, 1300 Morris Park Avenue, Room 504 Forchheimer Building, Bronx, NY, 10461, USA

**Keywords:** Trypanozoma cruzi, Chagas Disease, Adipocytes, Metabolism, Mitochondria, Latency

## Abstract

*Trypanosoma cruzi*, the etiologic agent of Chagas disease, causes a latent infection that results in cardiomyopathy. Infection with this pathogen is a major socio-economic burden in areas of endemic infection throughout Latin America. The development of chagasic cardiomyopathy is dependent on the persistence of this parasite in host tissues. Pathogenesis of this cardiomyopathy is multifactorial and research indicates that it includes microvascular dysfunction, immune responses to host and parasite antigens, and various vasoactive and lipid mediators produced by both the host and parasite. It has been demonstrated that *T. cruzi *persists in adipose tissue and uses fat as a nutritional niche in infected hosts. This chronic infection of adipose tissue plays an important role in the pathogenesis and persistence of this infection and involves mitochondrial stress responses as well as the production of various anti-inflammatory adipokines and pro-inflammatory cytokines by both white and brown adipose tissue. The changes in diet in endemic regions of infection have resulted in an epidemic of obesity that has significant implications for the pathogenesis of *T. cruzi *infection and the development of chagasic cardiomyopathy in infected humans.

## Introduction

Infection with *Trypanosoma cruzi* causes Chagas disease. Cardiomyopathy, due to *T. cruzi* infection, develops in about 30% of patients with Chagas disease and is a major socio-economic burden in Latin America ^1^. Evidence of *T. cruzi* infection has been found in mummies over 9000 years old from northern Chile and southern Peru ^2^. Chagas disease–related cardiomyopathy develops several years or decades after the initial infection. The pathogenesis of chronic Chagas cardiomyopathy is multifactorial ^3^. Mechanisms contributing to the progression of cardiomyopathy involve the persistence of parasites, myocardial damage, microvascular dysfunction, and neurogenic disturbances. Many of these changes are regulated by host immune-metabolic mediators. Although cardiomyopathy is the characteristic manifestation of chronic Chagas disease, this chronic infection can alter the function of other organs and tissues. One such organ is adipose or fat tissue ^4– 6^. Previously, we demonstrated that *T. cruzi* persists in adipose tissue and uses fat as a nutritional niche in infected murine models of *T. cruzi* infection and confirmed this finding in fat tissue from infected humans ^7^.

Adipose tissue is now recognized to function as an endocrine organ involved in whole body immune-metabolic homeostasis ^8, 9^. In mammals, three types of adipose depots commonly exist: white (WAT), brown (BAT), and beige/brite/brown-like (bAT) adipose tissues ^10^. These three types of adipose tissues differ in morphology (shape, color, number, and size of lipid droplets and number of mitochondria), development, localization, and functions ^11^. WAT and BAT have been extensively studied. WAT is a storage organ for energy in the form of lipids, whereas BAT regulates body temperature by producing heat via the expenditure of stored energy. Therefore, energy expenditure is greater in BAT compared with WAT. The morphology of white and brown adipocytes differs. In WAT, lipids are organized in a unique droplet (unilocular) and in BAT lipids are present in many droplets (multilocular). The size and number of mitochondria are significantly greater in BAT compared with WAT ^11^.

Adipose tissue is composed of adipocytes (80% of the cells) and a stromal fraction containing a variety of cells, including preadipocytes, adipose tissue–specific macrophages (ATM), T cells, endothelial cells, and nerve cells ^12^. The physiology of adipose tissue is highly regulated by the systemic energy homeostasis and vice versa. Importantly, fat tissue is highly sensitive to the whole body immune-metabolic challenges because of various infections, drug treatments, and disease states. Human hearts have epicardial and pericardial fat tissues, which may play a role in the pathogenesis of Chagasic cardiomyopathy. In this brief review, we summarize data on the role of adipose tissue and its dysfunction in the pathogenesis of chronic Chagas disease and in the progression of Chagasic cardiomyopathy.

## Adipose tissue: a reservoir for *T. cruzi* during infection

*T. cruzi* is an obligate intracellular protozoan that persists indefinitely following infection in mammals. For several decades, invasion mechanism(s) and persistence have been studied mainly in infected macrophages and cardiomyocytes ^13, 14^. In 1970, Shoemaker *et al*. first reported that *T. cruzi* may exist in BAT of infected mice ^15^. Using acute and chronic murine models of Chagas disease, our laboratory subsequently demonstrated that adipose tissue can serve as a reservoir for *T. cruzi* ^7^. Parasite load significantly increased in adipose tissues (both WAT and BAT) of *T. cruzi*–infected mice compared with heart tissue during early acute infection, suggesting that these parasites likely prefer lipid-rich fat cells over cardiomyocytes as a host cell environment ^7^. We also demonstrated the persistence of these parasites in adipose tissue during chronic cardiomyopathy, when parasite loads in the heart were significantly reduced ^7^. In humans, the persistence of this organism was demonstrated in adipose tissue in elderly seropositive patients with chronic chagasic heart disease ^16^.

Fat cells are a unique niche; they increase in number with age and generally are stable in adults. Although adipocytes die (about 10% of cells per year in an adult die), they are replaced by new cells. Adipocytes are rich in nutrients and secrete the anti-inflammatory adipokine adiponectin ^17, 18^. They therefore represent an excellent cell for persistence of an intracellular pathogen. Furthermore, in the adipose tissues of chronic *T. cruzi*–infected mice, there is a polarization of macrophages toward an M2 phenotype, probably further limiting sterilizing immunity and thereby allowing *T. cruzi* to persist in adipose tissue ^6^.

## Adipose tissue physiology during the acute and chronic stages of *T. cruzi* infection

*T. cruzi* infects both BAT and WAT ^7^. Interestingly, the parasite load was greater in both WAT and BAT of *T. cruzi*–infected mice compared with that found in the corresponding heart tissue at the early stages of acute infection (15 days after infection) ^7^. This time point of acute infection is characterized by a lack of blood parasitemia and overt illness. There is a significant change in adipose tissue physiology, even at this early stage of infection, and these changes differed in WAT and BAT ^4^. Adipose tissue physiology depends on the levels of adipogenesis and adipolysis (lipolysis), which are tightly regulated by the levels of localized adipokines, mainly peroxisome proliferator-activated receptor gamma (PPARγ) and adiponectin and adipose tissue–specific macrophage (ATM)-secreted tumor necrosis factor alpha (TNFα) ^19– 21^.

Adipocyte differentiation is controlled by a tightly regulated transcriptional cascade in which PPARγ and members of the C/EBP family are key players ^22^. *T. cruzi* infection significantly decreased the expression of PPARγ in BAT and significantly increased PPARγ levels in WAT at 15 days after infection and this was probably due to their different responses to infection-induced inflammation ^4^. Although there were significant similarities between BAT and WAT in regard to an increased infiltration of immune cells and elevated levels of lipolysis and inflammatory markers, WAT demonstrated a unique feedback response to elevated lipolysis by inhibiting nuclear factor kappa-light-chain-enhancer of activated B cells (NF-κB) activation ^4^. PPARγ was not decreased in the WAT during early acute infection ^4^. Levels of adiponectin were significantly reduced in both BAT and WAT, suggesting that deregulated lipid loss in adipose tissue may affect adiponectin levels independent of PPARγ levels ^4^.

By the end of the acute stage of infection (30 days after infection), mice displayed a threefold decrease in total body fat mass because of significant loss of lipid droplets compared with uninfected mice ^7^. The loss in lipid droplets was associated with significantly increased levels of infiltration of macrophages and inflammatory markers such as TNFα, interferon gamma (IFNγ), and interleukin 1 beta (IL-1β) in adipose tissue ^7^. The levels of adiponectin, an anti-inflammatory cytokine specifically secreted by adipose tissue, were significantly reduced by the end of acute infection ^7^. This is consistent with reported results of increased levels in serum of several inflammatory markers ^23^. Although TNFα and IFNγ were increased, they weakly correlated in the acute phase ^23^. The levels of inflammatory markers that were contributed mainly by adipose tissue, such as IL-6, monocyte chemoattractant protein-1 (MCP-1), and resistin, were significantly increased during acute infection in the murine *T. cruzi* infection model ^7^. Patients with the acute phase of Chagas disease also display increased circulating levels of IL-6 ^24^. Infants with congenital exposure to *T. cruzi* (diagnosed between 6 and 12 months of age) also have increased levels of IL-6 at 1 month of age ^24^. These data suggest that an intense inflammatory response in adipose tissue probably contributes to the circulating cytokine levels during acute infection.

The levels of body fat mass increased in *T. cruzi*–infected mice when they reached the chronic stage (90 days after infection) of infection compared with the acute stage of infection (30 days after infection); however, these levels of body fat were significantly lower than those of uninfected mice of the same age, suggesting that impaired fat tissue physiology and metabolism may occur in chronic *T. cruzi* infection. Levels of TNFα, IFNγ, and IL-1β in the adipose tissue of chronically infected CD1 mice significantly increased at 90 days after infection ^7^. Serum levels of several cytokines and chemokines (TNFα, IL-1β, IL-4, IL-5, CCL-5, CCL-11, CXCL-9, IL-2, IL-10, IL-17, and CCL-3) also increased during chronic infection in C57BL/6 mice (depending on the strain of *T. cruzi*) ^25, 26^. These levels of serum pro-inflammatory markers subside during the indeterminate stage and then increase during chronic stages in *T. cruzi*–infected humans with cardiac abnormalities ^27, 28^. Symptomatic chronic Chagas patients displayed greater levels of pro-inflammatory cytokines such as TNFα, IFNγ, and IL-6, and asymptomatic patients showed higher levels of IL-4, IL-10, IL-13, and transforming growth factor beta (TGFβ) ^27– 29^. There is a significantly increased loss of fat tissue in mice that develop cardiomyopathy compared with mice that do not develop cardiomyopathy during the chronic stage of infection ^7^ and adipose tissues from these mice expressed higher levels of TNFα, IFNγ, and IL-1β ^7^. These data suggest that adipose tissue physiology is altered during acute and chronic infections and adipose tissue–generated anti-inflammatory adipokines and pro-inflammatory cytokines contribute to the levels of serum inflammatory markers.

## Mitochondrial and endoplasmic reticulum stress during acute and chronic *T. cruzi* infection

Mitochondria play a significant role in crucial metabolic processes, including the tricarboxylic acid cycle, pyruvate decarboxylation, oxidative decarboxylation of fatty acids (β-oxidation), and degradation of branched amino acids. A primary function of mitochondria is to produce more than 95% of adenosine triphosphate (ATP) for cellular energy consumption ^30^. WAT adipocytes are classically large with a single big lipid droplet, and at the periphery of lipid droplets, a few mitochondria and tiny (but detectable) smooth endoplasmic reticulum (ER) ^11^. BAT adipocytes are small with multiple small lipid droplets surrounded by abundant mitochondria and little to no ER ^11^. Metabolic processes such as lipolysis and lipogenesis are strictly regulated in adipocytes and the mitochondria play a major role in this process ^11^. Lipolysis of triglycerides in lipid droplets results in the cytoplasmic accumulation of free fatty acids. Fatty acids can be translocated—through the carnitin acyl transferases—to the mitochondrial matrix, where they undergo β-oxidation, with the concomitant generation of acetyl coenzyme A (acetyl-CoA) and synthesis of ATP ^31^. Incomplete oxidation of accumulated fatty acids results in cell death due to lipotoxicity ^32^.

We have demonstrated that elevated levels of lipolysis of lipid droplets and loss of lipid contents (and adipocytes) are key factors in adipose tissue pathogenesis during acute *T. cruzi*–infected mouse models ^4^. The increased free fatty acids undergo further catabolism as demonstrated by increased levels of PPARα in adipose tissue ^4^. This increase in β-oxidation increases the cellular levels of reactive oxygen species (ROS) ^33^. In WAT adipocytes, which have relatively few mitochondria and very large lipid droplets compared to BAT, dysregulated lipolysis of lipid droplets during infection creates an immense burden on mitochondrial β-oxidation function and leads to elevated cellular ROS levels and mitochondrial oxidative stress. Continued stress on mitochondria during the acute stage of infection leads to significant ER stress and dysfunctional mitochondria functon, eventually resulting in cell death ^33^. Wen *et al*. demonstrated that both BAT and WAT display oxidative stress markers during acute and chronic *T. cruzi* infection; however, the pattern of intensity changes between BAT and WAT and acute and chronic stages of infection ^34^.

Adipose tissues from *T. cruzi*–infected mice demonstrate significantly increased mRNA levels of genes encoding components of NADPH oxidase complex, suggesting that the production of ROS might be enhanced in BAT and WAT during infection ^34^. In addition, mRNA levels of anti-oxidant markers such as glutathione peroxidase (GPX) and superoxide dismutase (SODs) are reduced in BAT and WAT, consistent with an increase in oxidative stress in acutely and chronically infected mice ^34^. Interestingly, mRNA levels of eosinophil peroxidases increase in both BAT and WAT during acute infection but decrease in BAT during chronic infection ^34^. This suggests that an eosinophil response may occur in both WAT and BAT.

**Figure 1.**
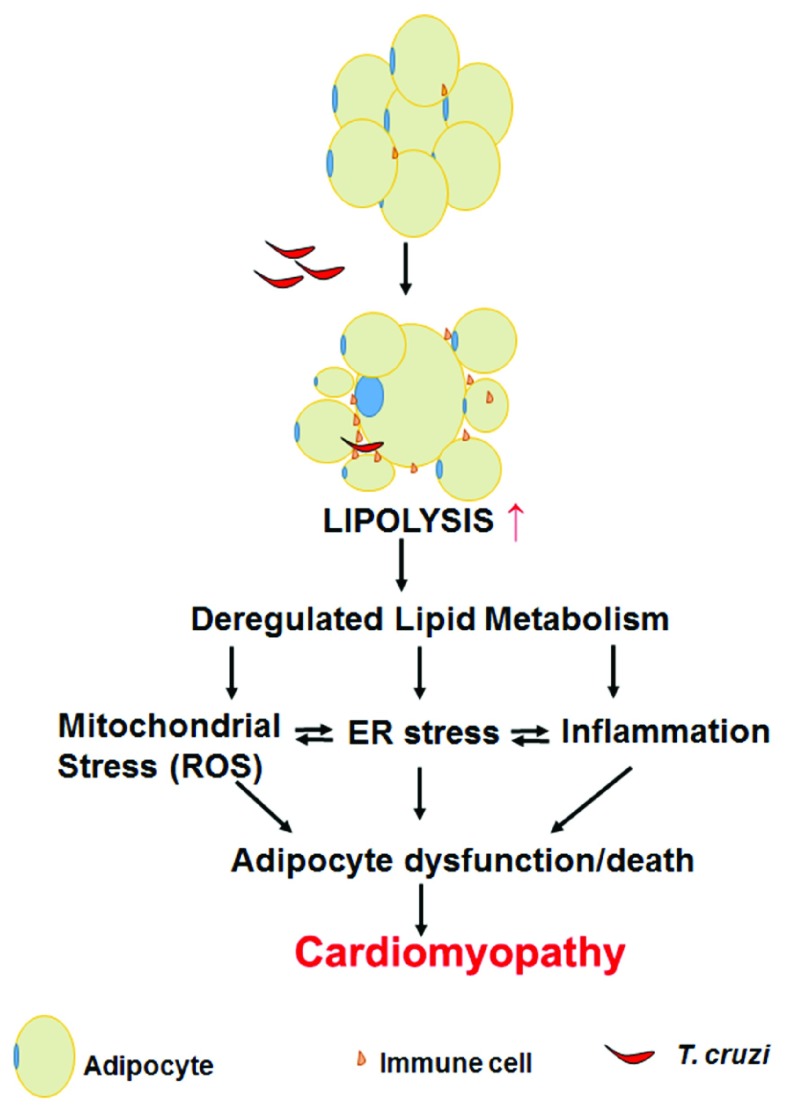
A pictorial representation of the effect of *Trypanosoma cruzi* infection on adipose tissue physiology. Infection causes adipocyte lipolysis, leading to mitochondrial oxidative stress, endoplasmic reticulum (ER) stress, inflammation, and a loss of fat cells. These pathologic changes contribute to the development of cardiomyopathy. ROS, reactive oxygen species.

WAT is under significant oxidative stress during acute *T. cruzi* infection which probably persists in chronic infection. This is supported by data showing increased levels of protein carbonylation and malonyldialdehydes in BAT and WAT during acute and chronic stages of infection, respectively, in a murine Chagas model ^34^. The levels of protein carbonylation and malonyldialdehyde are biomarkers of oxidative stress and lipid peroxidation, respectively. Increased ROS production and mitochondrial oxidative stress are associated with ER stress ^33^. In the adipocyte, the ER is directly involved with lipid droplet formation and the maintenance of lipid homeostasis ^35^. Adipocytes generally have scant ER and are highly susceptible to ER stress under conditions of deregulated lipolysis and oxidative stress ^36^. The combination of elevated ROS production and ER stress can easily trigger adipocyte apoptosis and cell death, leading to the observed loss of fat cells in the murine models of *T. cruzi* infection ( Figure 1).

## The impact of loss of adipose tissue on the pathogenesis of human Chagas cardiomyopathy

Murine models of chronic Chagas cardiomyopathy display an inverse relation between body fat mass and susceptibility to Chagas cardiomyopathy progression ^7^. The exact role of fat tissue and its immune-metabolic dysregulation in the pathogenesis of Chagas disease in patients with or without cardiomyopathy or heart failure is currently unknown; however, a few studies have evaluated body mass index (BMI) data in patients with various stages of Chagas disease. A study on beta-blocker therapy and mortality, reported that patients with Chagas heart failure displayed a significantly lower BMI compared with other non-Chagas heart failure patients (24.1 ± 4.1 versus 26.3 ± 5.1, *P* = 0.001) ^37^. Data (from the United Network for Organ Sharing database) collected from patients who were heart transplantation recipients between 1987 and 2015 in Brazil indicate that patients with Chagas cardiomyopathy had a significantly decreased BMI compared with idiopathic dilated cardiomyopathy patients (24 ^22– 26^ versus 26 ^23– 30^, *P* = 0.007) ^38^. In a study that reported on the mode of death due to progressive heart failure in Chagas patients compared with non-Chagas patients with heart failure, the BMI was significantly lower in Chagas patients with heart failure compared with non-Chagas patients (23.5 [21.3–26.4] versus 25.3 [22.5–29.0], *P* = 0.003) ^39^. Overall, these various studies provide human data which are consistent with observations in the murine model of Chagas cardiomyopathy; however, more clinical data with cross-sectional and longitudinal studies need to be collected to elucidate the role of fat tissue in patients with different degrees of Chagas cardiomyopathy.

## Limitations and Future direction

The changes in body fat mass content during *T. cruzi* infection depend on the strain of mouse, strain of *T. cruzi*, size of the inoculum used to induce infection, and stage of the disease. The findings that are described in this review may not be applicable in general to all mouse-parasite strains and to humans. Further studies are warranted to evaluate the role of adipose tissue in the pathogenesis of this infection, including assessing changes seen in Chagas patients with different degrees of cardiomyopathy.

## Conclusions

The pathogenesis of Chagas cardiomyopathy is complex and multifactorial. Adipose tissue is an immune-metabolic organ that has been demonstrated to play a significant role in the pathogenesis of Chagas cardiomyopathy in murine models, to be a niche and reservoir to *T. cruzi* in both human Chagas cardiomyopathy and murine models, and is an immune-metabolic regulator to the progression of Chagas cardiomyopathy in murine models. Adipocyte function in adipose tissue physiology depends mainly on its mitochondrial β-oxidation capacity. The number of mitochondria in white adipocytes is very small and thus mitochondrial β-oxidation stress induced by *T. cruzi* infection could easily result in a loss of adipocytes. Altered adipose tissue physiology, depending on its mitochondrial stress, complicates cardiac pathology, especially in the case of chronic Chagas disease. It is important to further investigate the pathophysiological role of adipose tissue and its signaling pathways in the progression of Chagas cardiomyopathy.
